# Predicting Subjective Usability from Kinematic Data in IMU-Based Robotic Teleoperation

**DOI:** 10.3390/s26155002

**Published:** 2026-08-06

**Authors:** Ionel Eduard Stan, Paolo Napoletano

**Affiliations:** Department of Informatics, Systems and Communication (DISCo), University of Milano-Bicocca, Viale Sarca 336, 20126 Milan, Italy; ioneleduard.stan@unimib.it

**Keywords:** robotic teleoperation, usability, inertial measurement unit (IMU), kinematic features, machine learning, NASA-TLX, user experience, Leave-One-Subject-Out, permutation test, SHAP

## Abstract

Robotic teleoperation is a core enabling technology spanning remote surgery, industrial inspection, and virtual-reality applications. Despite growing deployment, operator experience assessment still relies almost exclusively on post hoc subjective questionnaires, which preclude real-time monitoring and adaptive intervention. Here, the wearable IMU chain is considered not only as a command interface but also as an implicit sensing channel for operator state. We test whether end-effector kinematics generated by the IMU-to-robot mapping contain information about ten post-task workload and user-experience dimensions, comprising NASA-TLX-inspired workload scales together with usability, responsiveness, realism, intuitiveness, and perceived performance. A secondary analysis of a publicly available dataset (16 participants, 144 motion recordings, simulated UR10e arm) is conducted through a three-stage pipeline: bivariate correlation analysis (Pearson and Spearman), multivariate regression (10 model families, 16 feature-set combinations, Leave-One-Subject-Out validation), and binary classification (median-split). Statistical validity is assessed via 1000-permutation nested testing. Target-specific regression models reach $R^{2}\geq 0.50$ on seven out of 10 subjective dimensions, with a peak of $R^{2}=0.787$ for usability; permutation testing confirms significance for eight out of 10 targets. Binary classification achieves AUC ≥0.75 on nine out of 10 targets, with three dimensions reaching perfect AUC. SHAP analysis identifies temporal irregularity and distributional shape descriptors as the dominant kinematic explanatory families. These results support the feasibility of kinematics-based inference of operator experience and provide an offline proof of concept toward future real-time adaptive teleoperation systems.

## 1. Introduction

Robotic teleoperation—the remote control of robots by humans—is increasingly used in domains where direct human access is unsafe, impractical, or inefficient, including telesurgery [1], hazardous-environment manipulation, rehabilitation, and immersive robotic control [2,3,4,5]. Recent reviews emphasize that modern teleoperation research is no longer limited to communication and control stability, but also concerns interface embodiment, operator workload, shared autonomy, and human-state-aware adaptation [2,3].

For widespread adoption, the interaction must be fluid, intuitive, and non-fatiguing. A robot that users find frustrating, exhausting, or unnatural to control will ultimately fail [6]. Here, *user experience* (UX) refers to the operator’s overall perception of how natural, comfortable, and satisfying it is to interact with the system [7], making the measurement and optimization of subjective UX as critical as optimizing low-level control.

### 1.1. Limitations of Post Hoc Subjective Evaluation

The standard approach to UX assessment in teleoperation, and in human–computer interaction (HCI) more broadly, is the administration of questionnaires after task completion [8]. Instruments such as the NASA Task Load Index (NASA-TLX) [9], the System Usability Scale (SUS) [10], and the Questionnaire for User Interaction Satisfaction (QUIS) [11] are well-validated and widely deployed. Nevertheless, they share a fundamental limitation: being post hoc, they cannot capture the temporal dynamics of the experience, are susceptible to recency and memory biases [12], and do not enable real-time adaptive interventions.

### 1.2. Hypothesis and Research Questions

The central hypothesis motivating this work is that the *kinematics of the operator’s movement* during teleoperation encode information about the operator’s internal cognitive and affective state. We do not treat motion quality as evidence of expert competence. Rather, we test whether participant-level kinematic descriptors extracted from an inertial measurement unit (IMU)-based teleoperation interface contain statistical signal about self-reported workload and UX.

We operationalize this hypothesis through three research questions:

- *RQ1 (Correlation).* Do statistically significant linear and monotonic relationships exist between end-effector kinematic features and the ten subjective experience dimensions?
- *RQ2 (Regression).* Can supervised regression models predict the Likert-scale score of each dimension from kinematic features alone, generalizing to unseen users?
- *RQ3 (Classification).* When the problem is simplified to a binary high/low split, does predictive performance improve, and which dimensions become classifiable?

### 1.3. Contributions

From a sensing perspective, the same signal has two complementary functions: segment orientations captured by wearable IMUs are mapped into robot commands, while the resulting end-effector kinematics can be analyzed as an implicit indicator of operator workload and experience. The contribution of this study concerns the latter function. The main contributions of this paper are as follows:

1. A systematic correlation study (Pearson and Spearman) between 19 statistical, temporal, and spectral kinematic features and ten subjective UX metrics, with significance testing (*p*-values) for every coefficient.
2. A comprehensive regression benchmark comprising 10 models × 16 feature-set combinations × 10 targets, evaluated with strict Leave-One-Subject-Out (LOSO) cross-validation [13] and permutation-based significance testing.
3. A complementary binary-classification analysis (median-split) that reveals which UX dimensions become reliably separable from motion data when the granularity requirement is relaxed.
4. An interpretability analysis via SHAP (SHapley Additive exPlanations) [14] values, linking each prediction back to specific kinematic descriptors.
5. Public release of the analysis code to support reproducibility.
6. A clarified positioning of IMU-derived teleoperation kinematics as predictors of subjective UX and workload, distinct from task recognition, user identification, or questionnaire-only evaluation.

The remainder of the paper is organized as follows. Section 2 reviews the related literature. Section 3 describes the dataset, feature engineering, modeling pipeline, and statistical validation. Section 4 reports the experimental findings. Section 5 discusses findings, limitations, and implications. Finally, Section 6 concludes the paper and outlines future work.

## 2. Related Work

### 2.1. Robotic Teleoperation and IMU-Based Interfaces

Teleoperation systems comprise a human interface, communication layer, and remote or simulated robot executing the commanded action [2,3]. Master interfaces range from joysticks and haptic devices to vision-based, wearable, and extended-reality systems. Inertial measurement units (IMUs) remain attractive because accelerometers and gyroscopes embedded in wearable modules can track limb motion in real time with low cost, portability, and limited setup burden [15,16]. The dataset analyzed in this study originates from a system using three Bosch BNO055 IMUs (Bosch Sensortec GmbH, Reutlingen, Germany) mounted on the operator’s upper arm, forearm, and hand, transmitting quaternion data at 50 Hz to control a simulated Universal Robots UR10e arm (Universal Robots, Odense, Denmark) in Gazebo via ROS 2 [17].

### 2.2. Subjective Usability Evaluation

To evaluate such systems, well-established frameworks offer structured guidance. ISO 9241-11 [7] defines usability along the dimensions of effectiveness, efficiency, and satisfaction. The NASA-TLX [9] operationalizes perceived workload into six subscales (mental demand, physical demand, temporal demand, effort, frustration, and performance). Complementary instruments include the SUS for overall usability [10]. All these tools share the post hoc limitation discussed in Section 1, motivating the search for alternative assessment methods.

### 2.3. Movement Analysis and Affective/Cognitive State Inference

A growing body of work uses kinematic, inertial, and physiological signals to infer internal human states or to improve teleoperation control. Deep-learning approaches have been applied to facial expressions, speech, and body movement for affect recognition [18]. In the teleoperation domain, recent work has examined wearable IMU state estimation and rehabilitation teleoperation with IMU/sEMG sensing [5,16], while kinematic features have also been leveraged for *task recognition* [19]. These uses differ from decoding the operator’s *subjective experience*, illustrating the gap addressed here.

This gap extends into related fields. Usability evaluation in agricultural robotic teleoperation has been conducted via traditional questionnaires [20], and adaptive tutoring frameworks for human–robot interaction have been proposed [21], but these efforts do not attempt to *predict* subjective metrics from movement data.

### 2.4. Positioning of This Work

Several studies establish the sensing and robotic-control context for the present work. Skulj et al. [22] developed a wearable IMU interface that transforms upper-body orientations into collaborative-robot commands, including explicit calibration between the sensor, operator, and robot coordinate frames. Shintemirov et al. [23] presented an open-source wireless human-arm tracking system and demonstrated its application to real-time manipulator teleoperation. Zhu et al. [24] combined wearable IMU motion capture with individualized kinematic mapping and tremor attenuation. These studies primarily evaluate sensing architecture, motion reconstruction, or control performance. In contrast, the present work keeps the previously developed teleoperation pipeline fixed and investigates whether its IMU-derived end-effector trajectories provide a secondary sensing channel for operator workload and subjective experience.

To the best of our knowledge, no prior study has systematically investigated whether end-effector trajectory descriptors from IMU-based robotic teleoperation can predict the full range of NASA-TLX-inspired subjective workload dimensions together with additional UX scales (usability, realism, responsiveness, intuitiveness). Our novelty is therefore not a new teleoperation controller, task-recognition model, or questionnaire instrument; it is the secondary-analysis pipeline that links IMU-derived teleoperation kinematics to participant-level subjective UX and workload labels.

## 3. Materials and Methods

### 3.1. IMU-Based Teleoperation and Control Architecture

**Figure 1:**
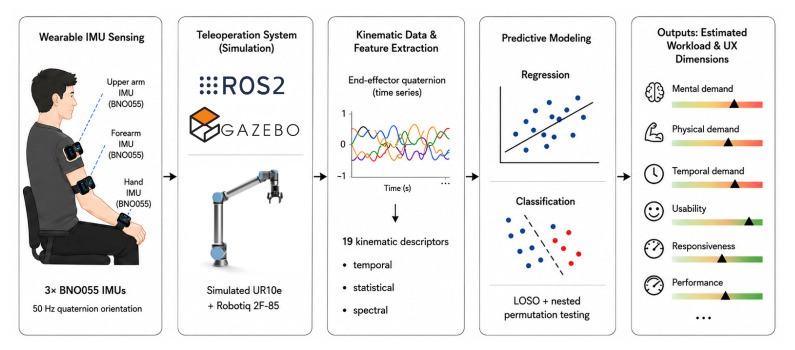
Overview of the IMU-based teleoperation and predictive-analysis pipeline. Three Bosch BNO055 IMUs, positioned on the operator’s upper arm, forearm, and hand, provide orientation quaternions at 50 Hz for controlling a simulated UR10e robot equipped with a Robotiq 2F-85 gripper through ROS 2 and Gazebo. End-effector quaternion trajectories are converted into 19 temporal, statistical, and spectral kinematic descriptors. Regression and binary-classification models are evaluated using Leave-One-Subject-Out validation and nested permutation testing to estimate ten subjective workload and user-experience dimensions. The indicators shown in the final block are illustrative examples of the complete set of prediction targets.

The experimental system underlying the analyzed dataset is summarized in Figure 1. The human-side sensing subsystem comprised three Bosch BNO055 inertial measurement units, interfaced through Arduino MKR1000 boards (Arduino, Monza, Italy) and mounted on the upper arm, forearm, and hand of each operator. The sensor nodes transmitted orientation quaternions at 50 Hz to a desktop ROS 2 system via MQTT. The ROS 2 modules used the segment orientations to compute the target end-effector pose, while a Cartesian motion controller interpolated the commanded motion of a Universal Robots UR10e equipped with a Robotiq 2F-85 gripper (Robotiq, Lévis, QC, Canada). Both the robot and gripper were simulated in Gazebo; no physical robot actuation was involved in the dataset analyzed here. A button integrated with the hand unit provided the gripper command. Further implementation and validation details of the original teleoperation platform are reported in [17,19].

The sensing chain therefore had two roles. First, the wearable IMUs generated the motion commands used for robot teleoperation. Second, the resulting kinematic streams constituted an implicit behavioral sensing channel describing how the operator interacted with the robot. The present work addresses this second role through a participant-level secondary analysis. It does not propose a new IMU device, motion-mapping method, or robot controller.

### 3.2. Dataset and Experimental Protocol

We performed a secondary analysis of the teleoperation dataset released by Fornaro et al. [17], with the goal of testing whether motion-derived kinematic descriptors can predict subjective user-experience dimensions. The released questionnaire metadata contain 16 male participants. Age bins were distributed as follows: 18–29 years, n=9; 30–39 years, n=6; and 40–49 years, n=1. Virtual-environment experience was mostly limited: no experience, n=6; low experience, n=4; average experience, n=3; and moderate experience, n=3. Thus, 10 of 16 participants (62.5%) reported no or low virtual-environment experience. Professional level, expert status, and prior teleoperation training history are not available as variables in the released dataset; consequently, the cohort should be interpreted as a mostly novice or low-experience sample, not as an expert benchmark. Three IMU sensors (Bosch BNO055 on Arduino MKR1000 boards) were attached to the upper arm, forearm, and hand of each participant, streaming orientation quaternions at 50 Hz to control a Universal Robots UR10e with a Robotiq 2F-85 gripper, simulated in Gazebo via ROS 2. An overview of the wearable sensing configuration, robotic teleoperation system, and predictive-analysis pipeline is shown in Figure 1. Each participant performed three manipulation tasks of increasing complexity, each repeated three times, yielding 16×3×3=144 motion recordings. For each trial, kinematic time-series were recorded for quaternion components (x,y,z,w) of the three IMU segments and the target end-effector pose. Post-task assessment was collected once per participant using a five-point Likert scale [25] (1 = strongly disagree, 5 = strongly agree) on ten targets: *physical_demand*, *mental_demand*, *temporal_demand*, *effort*, *frustration*, *usability*, *responsiveness*, *realistic*, *intuitive*, and *performance*. The all-male composition of the sample is acknowledged as a limitation (Section 5.4).

### 3.3. Feature Engineering and Data Preparation

For each kinematic signal, we extracted 19 statistical, temporal, and spectral descriptors: min, max, mean, median, std, variance, 4th_moment, 5th_moment, skewness, kurtosis, rms, iqr, total_sum, range, entropy, std_peaks, spectral_power, spectral_mean, spectral_median. The data treatment was supervised learning with kinematic descriptors as predictors and questionnaire responses as labels. Because questionnaire labels were available at participant level only, trial features were aggregated by arithmetic mean over each participant’s nine recordings, yielding one feature vector per participant and avoiding any artificial inflation of the label sample size. The predictive pipeline used end-effector quaternion descriptors only (ee_quat: axes x,y,z,w), giving 76 base predictors per user. Feature-set variants comprised all 15 non-empty subsets of {x,y,z,w} plus one avg set (axis-wise average across all four components), for 16 configurations per model. Likert responses were mapped to integers 1–5; for correlation analysis only, negatively valenced targets (physical/mental/temporal demand, effort, frustration) were sign-inverted so that higher values consistently denote better experience.

### 3.4. Bivariate Correlation Analysis

We computed Pearson (*r*) and Spearman (ρ) correlations between each feature and each target, with two-tailed significance testing. We report exact *p*-values and highlight associations satisfying both:

- A practical effect size threshold |r|≥0.30 and |ρ|≥0.30, and
- A statistical threshold p<0.05 for both Pearson and Spearman correlations.

This stage was exploratory and served to characterize the feature–target association structure before predictive modeling.

### 3.5. Multivariate Regression

We evaluated ten standard supervised regression families implemented through scikit-learn or XGBoost, rather than custom neural or classifier architectures [26,27]:

- LinearRegression,
- Ridge,
- Lasso,
- ElasticNet,
- KNeighborsRegressor,
- SVR,
- DecisionTreeRegressor,
- RandomForestRegressor,
- GradientBoostingRegressor, and
- XGBRegressor.

Hyperparameter grids are summarized in Table 1; selection was performed through inner-fold grid search.

Evaluation employed Leave-One-Subject-Out (LOSO) outer validation (16 folds) [13]: preprocessing was fitted on training participants only, inner model selection via three-fold cross-validation (shuffled, fixed seed), and out-of-fold predictions were aggregated across all 16 participants. Standardization was fold-local to prevent data leakage. The primary tuning metric was $R^{2}$ [28]; reported metrics included $R^{2}$, RMSE, and MAE.

### 3.6. Binary Classification

Each target was dichotomized at its sample median (high vs. low). We evaluated ten standard supervised classification families, again using established scikit-learn or XGBoost implementations rather than author-defined model architectures [26,27]:

- LinearDiscriminantAnalysis,
- RidgeLikeClassifier,
- LassoLikeClassifier,
- ElasticNetClassifier,
- KNeighborsClassifier,
- SVC,
- DecisionTreeClassifier,
- RandomForestClassifier,
- GradientBoostingClassifier, and
- XGBClassifier.

Table 2 summarizes the classification hyperparameter grids and fixed settings. The same LOSO outer protocol and inner three-fold tuning strategy were used. The primary tuning metric was ROC-AUC. Reported metrics include accuracy, balanced accuracy, macro-F1, and AUC.

### 3.7. Statistical Validation

After estimation, for each target the best target-specific configuration was subjected to nested permutation testing ($N_{\mathrm{perm}}=1000$, α=0.05): the full LOSO and tuning procedure was recomputed under each label permutation to reduce post-selection bias. Here, $s_{\mathrm{obs}}$ denotes the observed score of the selected model, $s_{k}$ denotes the score obtained under the *k*th label permutation, and $N_{\mathrm{perm}}$ is the number of permutations. Empirical *p*-values were defined as

(1) $$p=\frac{1+\#\{s_{k}\leq s_{\mathrm{obs}}\}}{N_{\mathrm{perm}}+1}\;\mathrm{for}\;\mathrm{RMSE}\;(\mathrm{lower}\;\mathrm{is}\;\mathrm{better}),$$

(2) $$p=\frac{1+\#\{s_{k}\geq s_{\mathrm{obs}}\}}{N_{\mathrm{perm}}+1}\;\mathrm{for}\;\mathrm{AUC}\;(\mathrm{higher}\;\mathrm{is}\;\mathrm{better}).$$

Bootstrap confidence intervals and global-vs-target-specific comparison tables were additionally computed as inference summaries.

This research design is therefore appropriate for a secondary, participant-level predictive analysis rather than for causal inference or deployment validation. The design deliberately combines a small-sample-aware outer LOSO protocol, fold-local preprocessing, target-specific model selection, and nested permutation testing so that every reported predictive effect is evaluated on held-out participants and then tested against label-permuted null models. At the same time, the design cannot support subgroup comparisons, expert-versus-novice conclusions, causal claims about movement quality, or threshold-optimized operational classifiers; these questions require a prospective study with larger and more diverse samples and per-trial or continuous labels.

### 3.8. Final Model Refit and Explainability

Best configurations were refit on the full data for each target to produce final model artifacts. Model explainability was then computed with SHAP [14] for all ten targets in both tracks. SHAP outputs were exported both as per-target figures and as a consolidated table.

All experiments were executed with a fixed random seed (42), LOSO outer validation, and 1000 nested permutations; the full analysis code is publicly released to support reproducibility (https://github.com/eduardstan/usability_teleop, accessed on 2 August 2026).

## 4. Results

### 4.1. Exploratory Data Analysis

#### 4.1.1. Questionnaire Response Distributions

**Figure 2:**
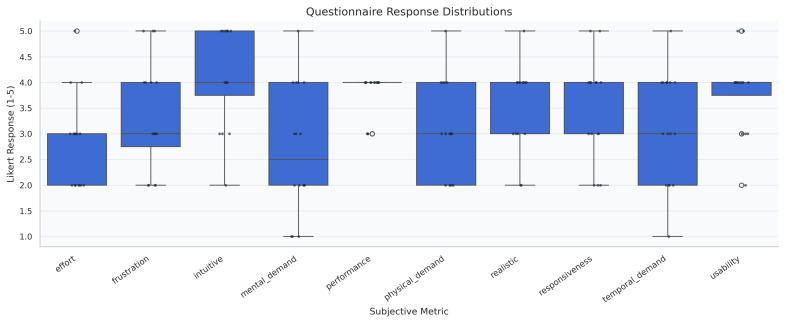
Distribution of questionnaire responses per metric. Median lines, boxes (IQR), and whiskers are shown.

Figure 2 shows score distributions across the 16 participants for all ten questionnaire metrics. *Performance* exhibits the tightest spread (median =4, IQR =[4,4], range =[3,4]), indicating consistently high perceived task completion quality. *Usability* also centers at four (median =4, IQR =[3.75,4]) but spans a wider range (2–5), suggesting inter-individual variability in interaction quality. In contrast, workload-oriented dimensions (physical demand, mental demand, temporal demand, effort, frustration) show broader dispersion, especially *mental_demand* (range 1–5), which provides useful variance for downstream modeling.

#### 4.1.2. Inter-Metric Correlations

**Figure 3:**
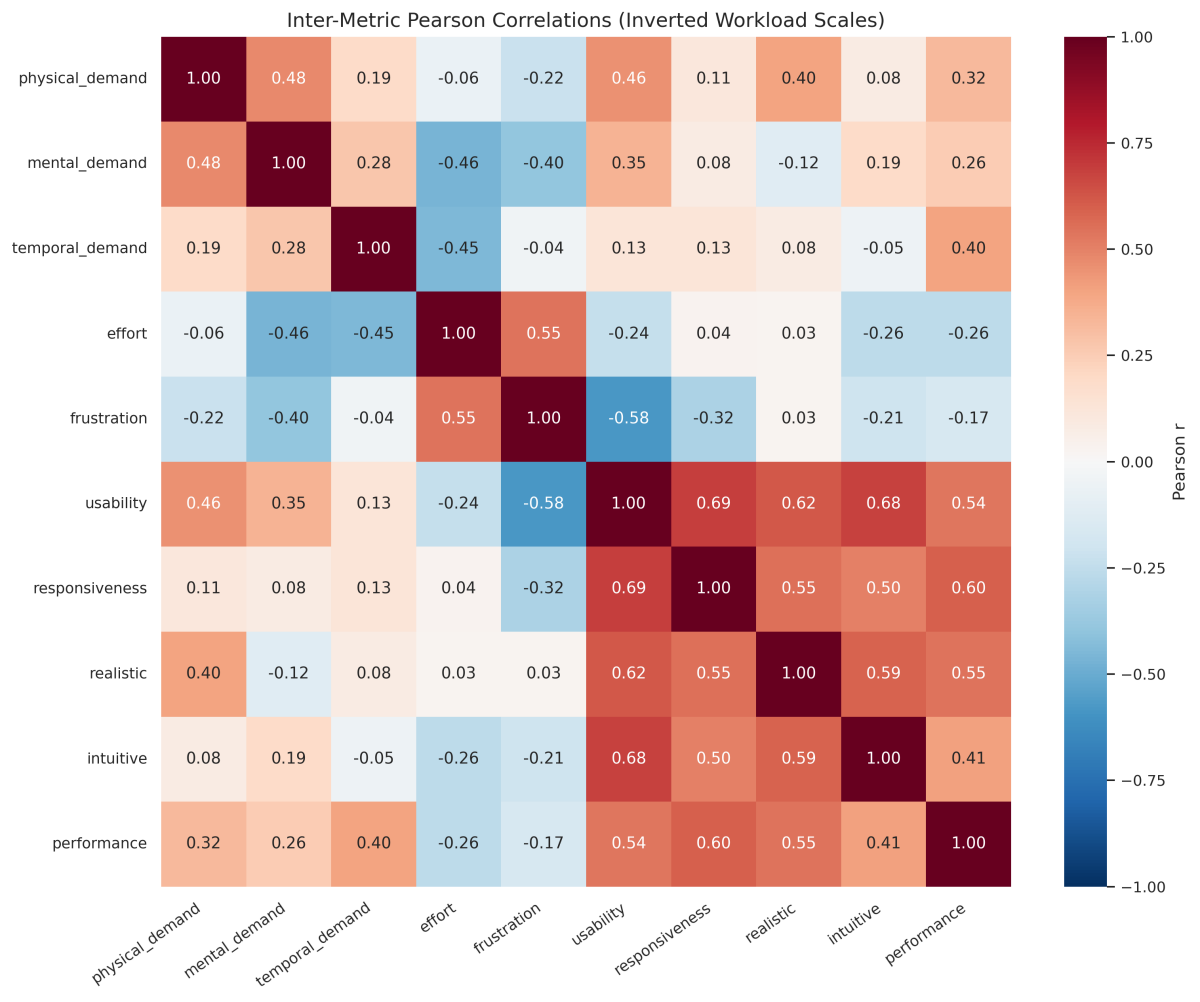
Correlation matrix among the 10 subjective metrics (higher = more positive experience for all metrics after inversion).

A Pearson correlation matrix among the ten questionnaire dimensions (with inversion of negatively valenced workload items) reveals two structurally distinct blocks, as shown in Figure 3.

The first block groups workload-related dimensions: *physical_demand*, *mental_demand*, and *temporal_demand* are mutually positively correlated (e.g., r=0.48 for physical–mental, r=0.28 for mental–temporal), reflecting a shared cognitive and physical load component. *Effort* and *frustration* form a tighter sub-cluster within this block (r=0.55), consistent with their conceptual overlap in NASA-TLX.

The second block comprises the positive-experience dimensions {*usability*, *responsiveness*, *realistic*, *intuitive*, *performance*}, which are consistently positively correlated (mean pairwise r=0.57, min r=0.41, max r=0.69). The strongest pairs are *responsiveness*–*usability* (r=0.69) and *intuitive*–*usability* (r=0.69), followed by *realistic*–*usability* (r=0.62), suggesting that perceived naturalness, responsiveness, and control quality converge into a unified experience of usability.

The two blocks are antagonistically coupled: *frustration* vs. *usability* (r=−0.58), *frustration* vs. *responsiveness* (r=−0.32), and inverted *effort* vs. inverted *mental_demand* (r=−0.47). This structure indicates that workload and positive-experience dimensions are separable but interacting factors, which motivates treating each subjective target independently in the predictive modeling stage.

### 4.2. Correlation Analysis

Table 3 reports the strongest *end-effector quaternion* correlations between kinematic descriptors and subjective targets. Entries are restricted to associations satisfying *both* (i) practical effect size (|r|≥0.30 and |ρ|≥0.30); and (ii) statistical significance (Pearson $p_{r}<0.05$ and Spearman $p_{\rho }<0.05$).

A robust pattern emerges around *frustration*: several end-effector descriptors (entropy, higher-order moments, variance) show consistent positive associations, while mean_ee_quat.w and std_peaks_ee_quat.x are negatively associated. This indicates that both distributional shape and temporal irregularity of motion are linked to perceived frustration. For *mental_demand*, the strongest end-effector association is kurtosis_ee_quat.y (r=−0.57, $p_{r}=0.021$; ρ=−0.60, $p_{\rho }=0.014$), consistent with the interpretation that more abrupt, heavy-tailed motion profiles are associated with higher cognitive load. For *realistic*, kurtosis_ee_quat.x is positively associated (r=0.59, ρ=0.60), suggesting that moderate kinematic variability and non-Gaussian movement structure are perceived as more natural.

**Figure 4:**
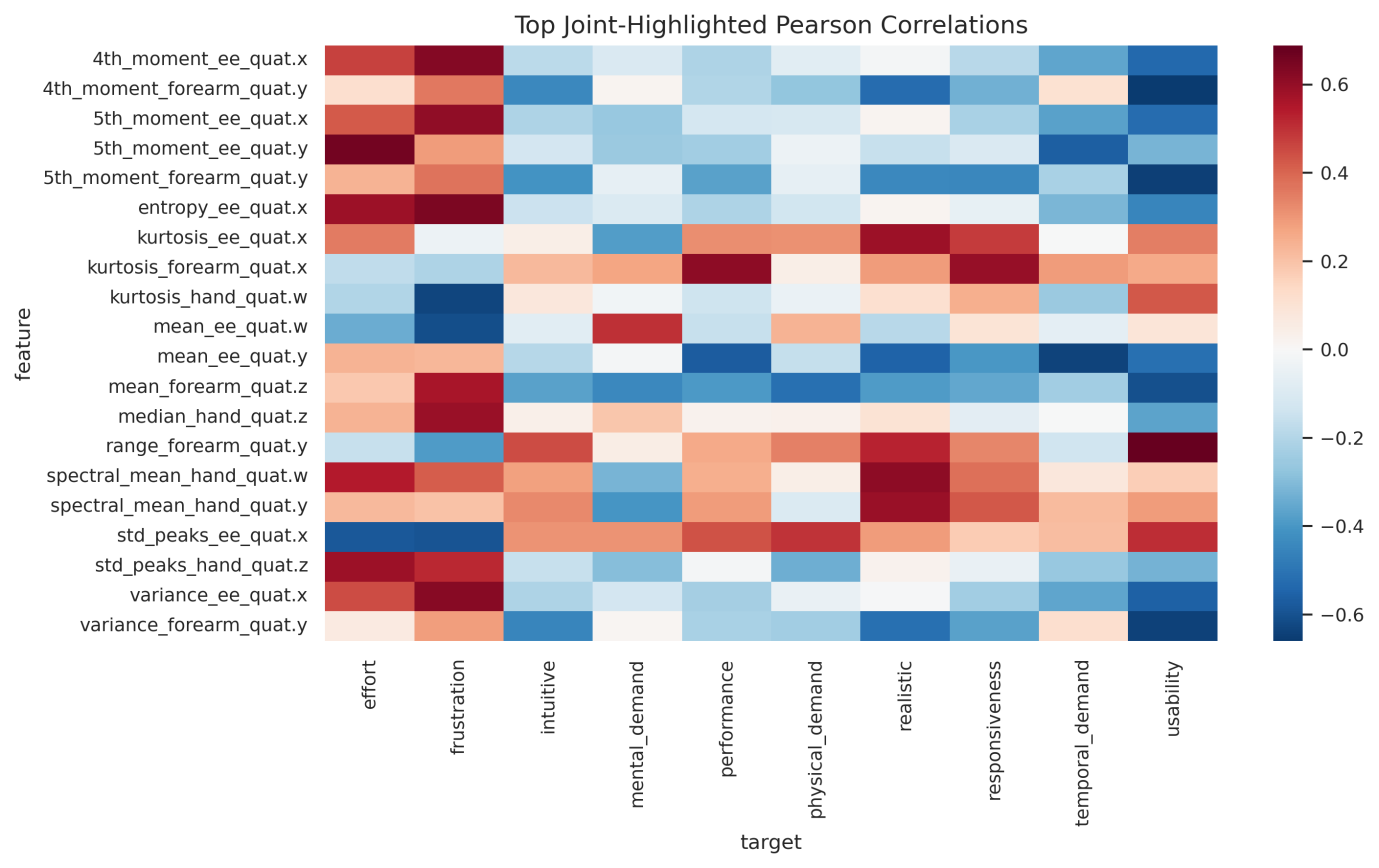
Pearson heatmap of joint-highlighted feature–target associations.

**Figure 5:**
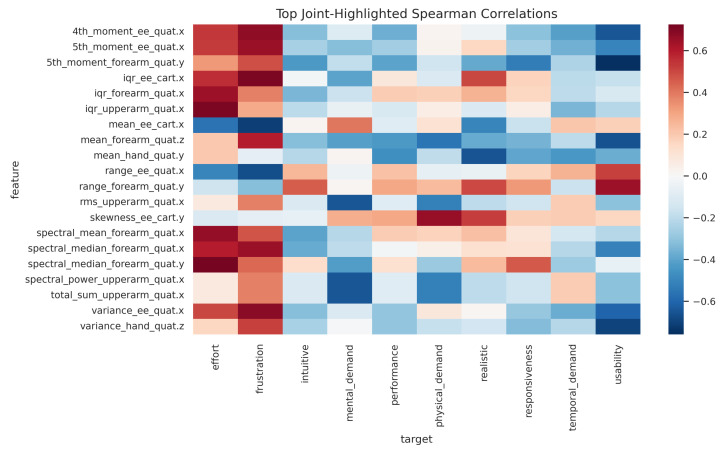
Spearman heatmap of joint-highlighted feature–target associations.

Taken together, the Pearson and Spearman analyses are directionally consistent across the highlighted associations. This agreement indicates that the detected feature–target relationships are not driven only by strict linear assumptions, but are also preserved under rank-based dependence. As shown in Figure 4 and Figure 5, the same targets and feature families recur across both maps, reinforcing the robustness of the observed correlation structure.

### 4.3. Regression Results

#### 4.3.1. Global Configuration Ranking

Regression estimation was executed with 10 model families and 16 feature-set configurations for each of the 10 targets, for a total of 10×16×10=1600 LOSO-evaluated model–feature-set–target runs. We use model_name | feature-set to denote the setting where the model model_name was trained on the feature-set feature-set.

**Figure 6:**
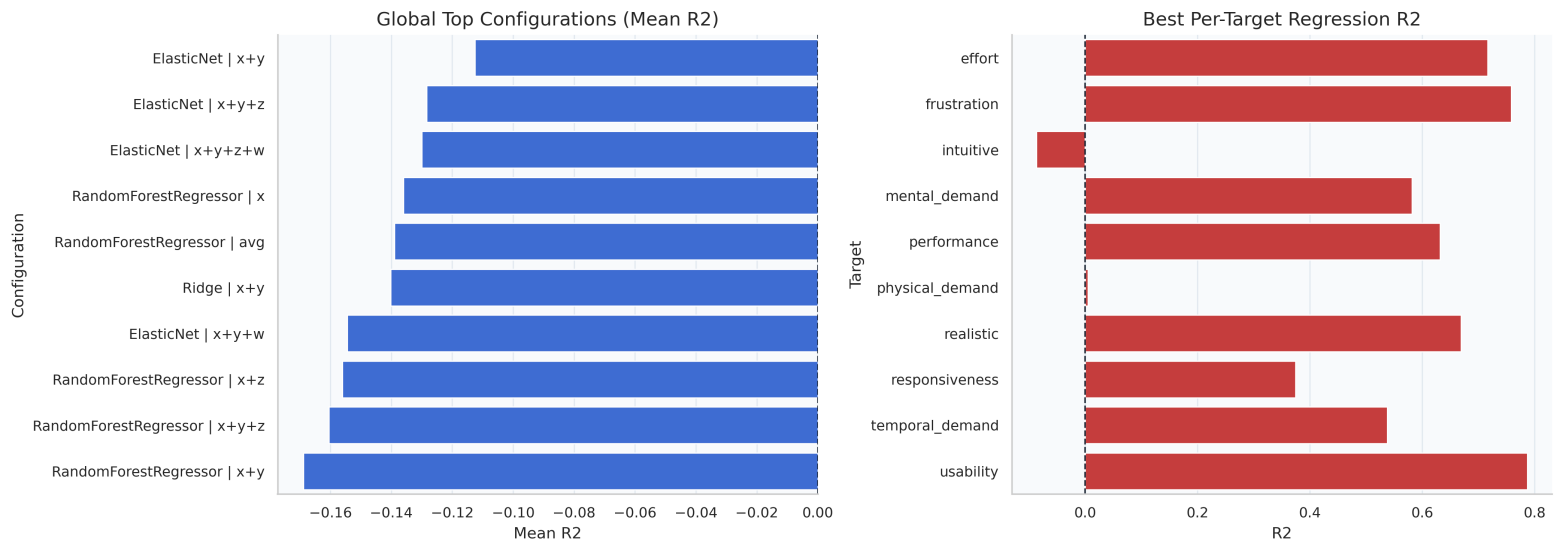
Regression overview: global top configurations (mean $R^{2}$) and best per-target $R^{2}$.

To quantify a *global* configuration, we averaged $R^{2}$ across targets for each (model, feature-set) pair. None of the 160 global configurations achieved a positive mean $R^{2}$: the best was ElasticNet | x + y scoring −0.113, followed by ElasticNet | x + y + z (−0.128) and ElasticNet | x + y + z + w (−0.130). The worst global configurations were mostly single-axis LinearRegression variants (especially axis w), with very large negative mean $R^{2}$, indicating unstable cross-target behavior in this small-sample setting. Figure 6 summarizes this contrast between weak global averages and strong target-wise optima.

#### 4.3.2. Target-Specific Performance

When model and feature-set selection was performed per target, performance improved substantially. Table 4 reports the best target-specific configuration under LOSO validation. Quantitatively, seven out of 10 targets reach $R^{2}\geq 0.50$, eight out of 10 reach $R^{2}\geq 0.30$, and only *intuitive* remains clearly non-predictable (negative $R^{2}$). The best-performing target is *usability* ($R^{2}=0.787$), followed by *frustration* ($R^{2}=0.758$) and *effort* ($R^{2}=0.716$).

#### 4.3.3. Global vs. Target-Specific Gap

**Figure 7:**
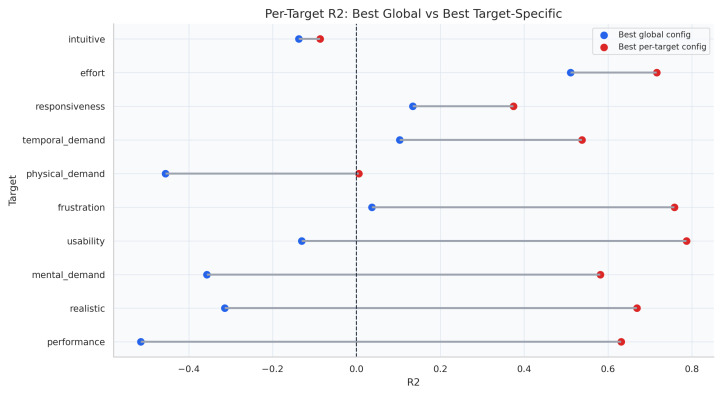
$\Delta R^{2}$ between best target-specific models and the best global regression configuration.

The global-to-local comparison confirms that a single shared regression configuration is suboptimal. As shown in Figure 7, using ElasticNet | x + y as global baseline, the target-specific best model improves $R^{2}$ for *all* targets: $\Delta R^{2}$ ranges from 0.051 (*intuitive*) to 1.146 (*performance*), with mean 0.610 and median 0.592. The largest gains are observed for *performance*, *realistic*, *mental_demand*, and *usability*, supporting a strongly target-dependent modeling strategy.

#### 4.3.4. Permutation Validation of Regression Effects

Nested permutation testing ($N_{\mathrm{perm}}=1000$) was applied to the best target-specific regression configuration for each target. Table 5 reports observed RMSE, null-distribution mean RMSE, and empirical *p*-values. Eight out of ten regression targets are significant under permutation testing at α=0.05, with only *physical_demand* and *intuitive* remaining non-significant. Notably, the non-significant pair corresponds to the two weakest targets in predictive fit ($R^{2}=0.005$ and $R^{2}=-0.087$, respectively), increasing internal consistency between effect size and statistical validation.

### 4.4. Binary Classification Results

#### 4.4.1. Global Configuration Ranking

Classification estimation was executed over 10 model families and 16 feature-set configurations for each of the 10 targets, i.e., 10×16×10=1600 LOSO-evaluated runs.

**Figure 8:**
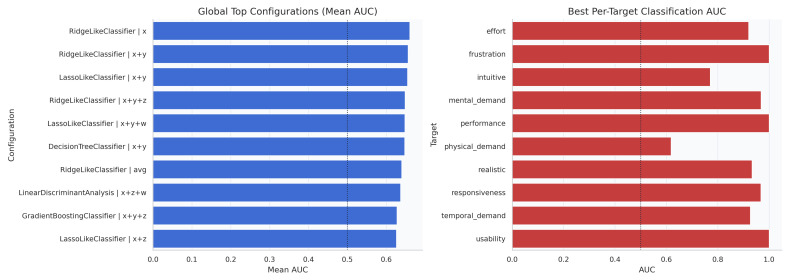
Classification overview: global top configurations (mean AUC) and best per-target AUC.

Global ranking was computed by averaging AUC across targets for each (model, feature-set) pair. The best global configuration was RidgeLikeClassifier | x with mean AUC =0.660, followed by RidgeLikeClassifier | x + y (0.656) and, in third place, LassoLikeClassifier | x + y (0.655). In contrast, SVC global variants were consistently weak in this setting (lowest mean AUC values). Figure 8 shows the global top configurations together with the best per-target AUC values.

#### 4.4.2. Target-Specific Performance

As in regression, target-specific tuning provided the strongest results. Table 6 reports the best classifier per target. The target-wise profile is strong in AUC terms: nine out of 10 targets reach AUC ≥0.75, and eight out of 10 exceed AUC ≥0.90. Three targets—*frustration*, *performance*, and *usability*—achieve perfect or near-perfect AUC. The *mental_demand* classifier follows this strong target-specific pattern, with AUC = 0.969 and matched accuracy, balanced accuracy, and macro-F1 of 0.875. Overall, the classification results indicate reliable high/low discrimination for most subjective dimensions, with the clearest limitation remaining *physical_demand*.

#### 4.4.3. Global vs. Target-Specific Gap

**Figure 9:**
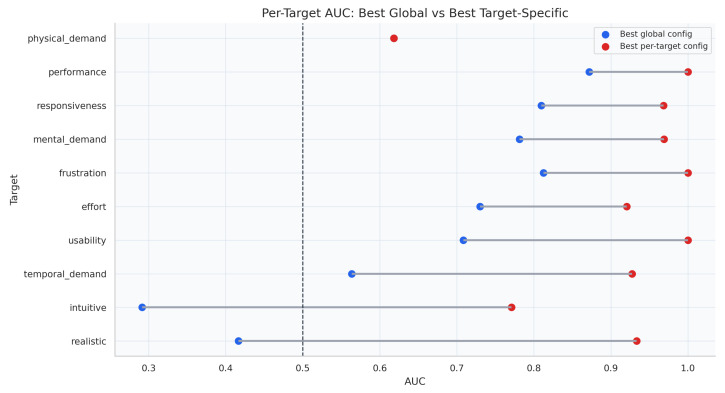
AUC gain of best target-specific classifiers over the best global classification configuration.

The global-vs-local comparison confirms systematic gains from target-specific modeling. Using RidgeLikeClassifier | x as global baseline, target-specific optimization improves AUC for nine out of 10 targets, with no change for *physical_demand*. Finally, ΔAUC ranges from 0.000 to 0.517 (mean 0.250; median 0.189), with the largest gains on *realistic*, *intuitive*, and *temporal_demand*. Figure 9 visualizes this per-target improvement profile.

#### 4.4.4. Permutation Validation of Classification Effects

**Figure 10:**
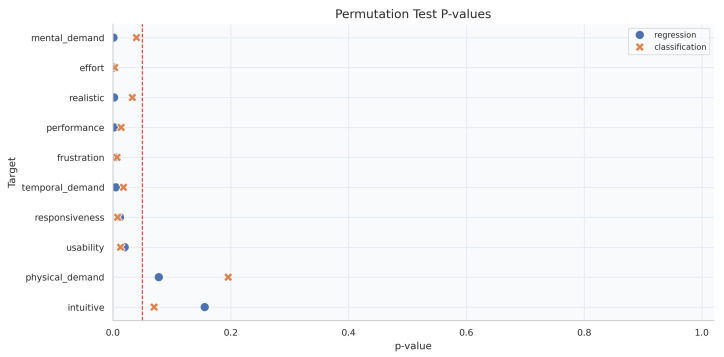
Permutation *p*-value summary across tracks; both regression and classification distributions are concentrated toward low *p*-values, consistent with the significant targets.

Nested permutation testing ($N_{\mathrm{perm}}=1000$) was run for the best classifier of each target. Table 7 reports observed AUC, permutation-null mean AUC, and empirical *p*-values. Thus, eight out of 10 classification targets are significant at α=0.05. The two non-significant targets (*intuitive*, *physical_demand*) are the same pair observed as weakest in the regression analysis and in the broader predictive profile. Figure 10 provides the aggregate permutation-view context (classification and regression distributions together), showing the *p*-value concentration toward low values for both tracks.

### 4.5. Feature-Set Impact Across Regression and Classification

**Figure 11:**
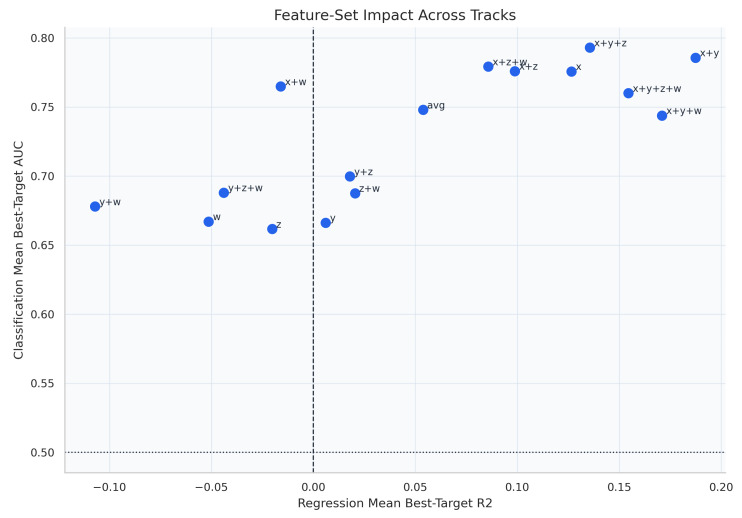
Feature-set impact across tracks: regression mean best-target $R^{2}$ vs. classification mean best-target AUC.

Beyond per-target model selection, we analyzed how each axis-combination feature set behaves *across tracks*. For each feature set, we computed the following: (i) the mean best-target regression score (${\bar{R^{2}}}_{\mathrm{best}}$); and (ii) the mean best-target classification score (${\bar{\mathrm{AUC}}}_{\mathrm{best}}$), aggregated over the ten targets. Figure 11 summarizes this two-track profile. The horizontal axis reports regression mean best-target $R^{2}$, the vertical axis reports classification mean best-target AUC, and each point is one feature set.

The first finding is that multi-axis sets dominate single-axis sets. The top regression set is x + y (${\bar{R^{2}}}_{\mathrm{best}}=0.187$), while the top classification set is x + y + z (${\bar{\mathrm{AUC}}}_{\mathrm{best}}=0.793$). In contrast, single-axis y, z, and w are among the weakest options in both tracks. A second finding concerns cross-track consistency: feature sets that perform well in regression tend also to perform well in classification, with Pearson correlation r=0.786 between ${\bar{R^{2}}}_{\mathrm{best}}$ and ${\bar{\mathrm{AUC}}}_{\mathrm{best}}$ across the 16 sets. This supports the view that a common kinematic information structure drives both continuous and dichotomized formulations. A third finding concerns stability: the avg set is not top-ranked in either track (${\bar{R^{2}}}_{\mathrm{best}}=0.054$, ${\bar{\mathrm{AUC}}}_{\mathrm{best}}=0.748$), but it remains competitive with relatively moderate dispersion across targets, suggesting a useful compromise when compact representations are preferred.

Table 8 reports the leading and trailing feature sets according to both tracks. Taken together, Figure 11 and Table 8 indicate that compact multi-axis combinations (especially x + y and x + y + z) offer the best trade-off across tracks, while single-axis sets are generally less robust. This analysis complements the per-target results of Section 4.3 and Section 4.4 by providing representation-level design guidance.

### 4.6. SHAP Feature-Importance Analysis

**Figure 12:**
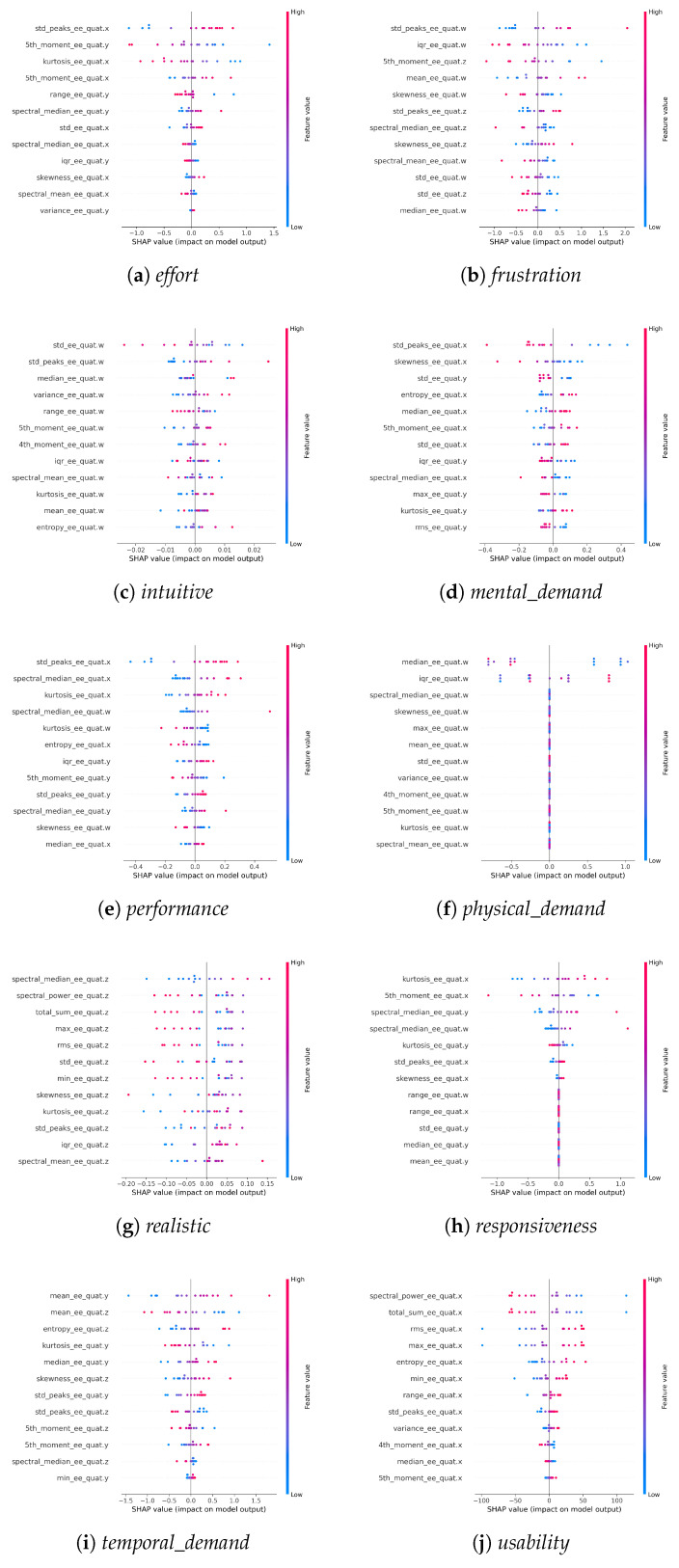
Regression SHAP summaries for all ten targets (5 × 2 grid).

**Figure 13:**
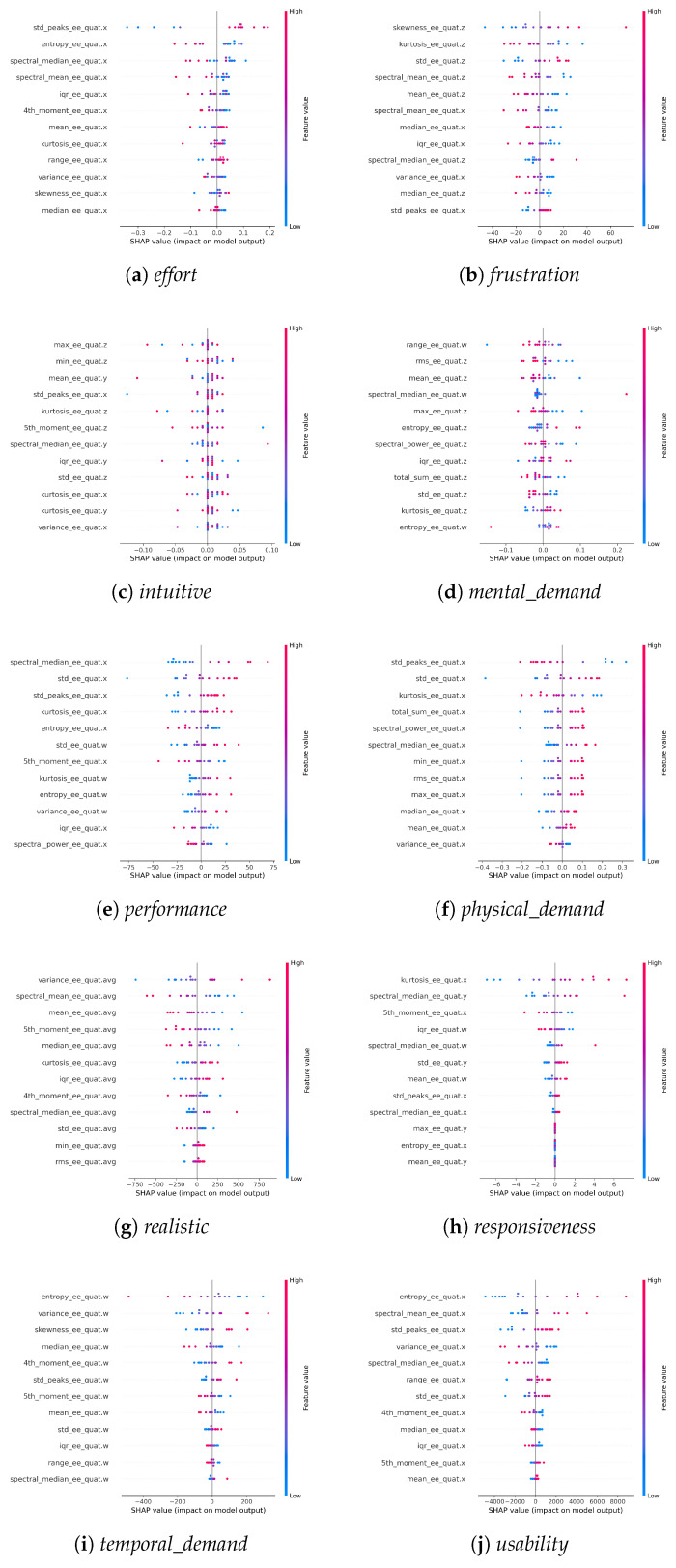
Classification SHAP summaries for all ten targets (5 × 2 grid).

To interpret the fitted target-specific models, we computed SHAP explanations [14] for *all* questionnaire dimensions in both tracks. This yields 20 panels in total (10 for regression and 10 for classification). For readability, we group them into two composite figures: regression (Figure 12) and classification (Figure 13). SHAP magnitudes are model- and scale-dependent; therefore, comparisons should be made primarily *within each panel* and by recurring feature families across targets, rather than by absolute value across different panels.

#### 4.6.1. Regression SHAP Patterns

Figure 12 shows the ten regression SHAP summaries. Three stable motifs emerge:

- **Temporal irregularity and higher-order moments** (e.g., std_peaks, kurtosis, 5th_moment) repeatedly appear in *effort*, *frustration*, *mental_demand*, and *responsiveness*.
- **Spectral descriptors** become dominant for targets such as *realistic* and *usability*, especially through spectral_power and spectral_median terms.
- **Axis-specific sensitivity** is preserved: several targets are driven by compact axis subsets (e.g., z for *realistic*, w for *physical_demand*, x + y + w for *performance*).

These trends are coherent with the regression accuracy profile reported in Section 4.3: targets with stronger predictive fit are also those with sharper, more structured SHAP signatures.

#### 4.6.2. Classification SHAP Patterns

Figure 13 reports the analogous ten-panel classification view. The main findings are as follows:

- **Consistency with regression**: Variability and shape descriptors (entropy, variance, higher-order moments, std_peaks) remain recurrent explanatory drivers.
- **Strong discriminative structure for high-AUC targets**: Targets such as *frustration*, *performance*, *usability*, and *responsiveness* show concentrated high-impact features, in line with their strong classification performance and permutation significance (Section 4.4).
- **Target-specific representation changes**: For *realistic*, the selected avg feature set yields axis-aggregated descriptors among the dominant SHAP contributors, whereas other targets remain axis-specific.

Overall, Figure 12 and Figure 13 provide a mechanistic complement to the predictive results: predictive signal is not uniformly distributed, but concentrated in interpretable kinematic families related to motion variability, temporal irregularity, and distributional shape.

## 5. Discussion

### 5.1. Answering the Research Questions

RQ1 (Correlation).

Yes, statistically significant correlations exist between end-effector kinematic descriptors and multiple subjective experience dimensions. The strongest individual associations involve *frustration*, with five end-effector features reaching |r|≥0.59 and |ρ|≥0.58 (Table 3). In addition, entropy and higher-order moments (4th_moment, 5th_moment) of the quaternion trajectory show consistent positive associations with *frustration* and *effort*, while mean_ee_quat.w is negatively associated. For *mental_demand*, kurtosis_ee_quat.y exhibits r=−0.57, ρ=−0.60 (p<0.025), indicating that more abrupt, heavy-tailed motion profiles co-occur with higher perceived cognitive load. Two coherent interpretive patterns emerged: (i) greater movement variability and temporal irregularity correlate with worse subjective experience (higher workload, more frustration), and (ii) moderate non-Gaussian kinematic structure (kurtosis) correlates with higher perceived realism (r=0.59, ρ=0.60). Both Pearson and Spearman analyses are directionally consistent, confirming that the detected associations are robust to strict linearity assumptions.

RQ2 (Regression).

Yes, substantially. No single global configuration achieved a positive mean $R^{2}$ across all ten targets, confirming that a one-size-fits-all model is inadequate for the heterogeneous set of subjective dimensions. However, when model and feature-set selection was performed per target, performance improved markedly: seven out of 10 targets reached $R^{2}\geq 0.50$, with the highest value being $R^{2}=0.787$ for *usability* (Table 4). Among workload-related targets, *effort* ($R^{2}=0.716$), *mental_demand* ($R^{2}=0.582$), and *temporal_demand* ($R^{2}=0.538$) all exceeded the 0.50 threshold. Critically, nested permutation testing confirmed statistical significance for eight out of 10 regression targets at α=0.05 (Table 5). Only *physical_demand* (p=0.078) and *intuitive* (p=0.156) remained non-significant—the same two targets that also showed the weakest predictive fit ($R^{2}\leq 0.005$), lending internal consistency to the results.

RQ3 (Classification).

Recasting the problem as binary classification (median-split) substantially strengthened the predictive profile: nine out of 10 targets achieved AUC ≥0.75, with three targets—*frustration*, *usability*, and *performance*—reaching perfect AUC of 1.000 (Table 6). Nested permutation testing confirmed significance for eight out of 10 targets at α=0.05 (Table 7), again with *physical_demand* and *intuitive* as the sole non-significant pair. This supports an offline proof-of-concept conclusion: when the granularity requirement is relaxed from exact Likert-score prediction to high/low discrimination, kinematic features contain statistically validated discriminative signal for most, but not all, UX dimensions under the submitted validation protocol.

### 5.2. Practical Implications

The results suggest that an adaptive teleoperation system equipped with a lightweight classifier monitoring end-effector kinematics in real time could

- Detect when an operator is experiencing high workload (e.g., elevated frustration or mental demand) and trigger assistive interventions such as motion smoothing, haptic guidance, or task simplification.
- Provide continuous, objective UX monitoring during system prototyping, reducing dependence on post hoc questionnaires and enabling experience-aware design iteration.
- Enable personalization by adapting control parameters (e.g., sensitivity, filtering) to the individual operator’s motion signature, building on the authenticated teleoperation framework introduced in [19].
- Serve as a probabilistic UX indicator for shared-control or adaptive-autonomy systems, where the control policy can reduce operator burden while preserving human authority. Turco et al. provide a concrete teleoperated-swarm example: their data-driven shared-control approach reduced NASA-TLX workload relative to conventional teleoperation while improving task performance [29]; related shared-control work also reports effects on cognitive load and trust [30].

The intended use is diagnostic and assistive: models should flag probable workload or UX states, not classify operator competence or penalize unfamiliar but effective movement styles.

The strong performance of simple linear models on several targets further supports deployment feasibility. In particular, the low computational footprint of these models makes them natural candidates for edge inference directly on wearable IMU hardware. Recent work has demonstrated that lightweight convolutional architectures can perform inertial gesture recognition on microcontroller-class edge devices [31], suggesting a realistic pathway for embedding real-time UX inference into the IMU nodes themselves, without offloading computation to a remote host.

### 5.3. Comparison to Related Work

Direct comparison with the prior literature is difficult because, to our knowledge, no existing study attempts to predict the full NASA-TLX workload profile from teleoperation kinematics alone. Stan et al. [19] used similar kinematic features for *task and user recognition* (a classification problem with different targets), achieving high accuracy; our work extends their feature-extraction pipeline to a fundamentally different question—inferring subjective experience rather than task identity. Adamides et al. [20] evaluated agricultural teleoperation usability via questionnaires alone, without attempting kinematic prediction. Recent IMU-based teleoperation studies have focused on state estimation, mapping, or rehabilitation-control use cases [4,5,16]; our contribution is complementary because it uses the recorded kinematics as predictors of subjective UX and workload labels. The interpretive motifs identified in Section 4.2—movement irregularity correlating with negative experience, and moderate complexity correlating with realism—align with broader findings in affective computing, where motion jerkiness has been linked to cognitive load and stress [18,32].

Related studies also demonstrate why such an operator-state signal could be useful. Roldán et al. [33] evaluated how immersive and predictive interfaces affected workload and situational awareness in multi-robot supervision. Ding et al. [34] and Styler et al. [35] evaluated vision-guided shared control for assistive manipulation, reporting improvements in task performance and, in the larger evaluation, reductions in NASA-TLX workload. Those studies manipulate the interface or allocation of control and subsequently measure operator outcomes. The present study addresses a complementary problem: estimating those outcomes from the motion produced during interaction. Accordingly, the proposed models should be understood as offline candidate estimators that could eventually inform shared-control interventions, rather than as evidence that a closed-loop intervention has already been validated.

### 5.4. Limitations

1. **Sample size.** The primary limitation is N=16 participants, and the participant-level aggregation described above leaves only 16 supervised rows for model fitting. It reduces the power of permutation tests and makes complex models prone to overfitting. While eight out of 10 targets survived strict nested permutation testing, a larger cohort would enable more powerful statistical analyses and the use of deeper models.
2. **Gender homogeneity.** All participants were male, which limits generalizability to mixed-gender populations. Gender-related differences in motor strategy [32] could affect both kinematic features and subjective ratings.
3. **Post hoc questionnaire and aggregation.** A single questionnaire administered after all nine trials introduces recency bias [12] and collapses temporal dynamics. Participant-level aggregation was necessary because labels were participant-level, but it removes trial-to-trial variation and prevents the analysis of within-session adaptation. Per-trial or continuous annotation would increase the effective sample size and enable temporal modeling.
4. **Experience and professional metadata.** The released dataset contains virtual-environment experience bins but not professional level, expert status, or expert benchmark trials. Therefore, the present models cannot support claims about expert performance, competence detection, or movement efficiency relative to professional operators.
5. **IMU calibration and drift.** The secondary dataset documents the recorded IMU/quaternion streams but does not provide enough calibration, filtering, or drift-correction metadata to quantify orientation drift retrospectively. One participant comment explicitly mentions drift, so calibration uncertainty should be treated as a limitation of the available secondary data rather than as a solved preprocessing issue.
6. **Biomechanical variability.** Different operators can accomplish the same teleoperation goal with different limb lengths, movement habits, postures, and path strategies. IMU-based UX models may therefore produce false-positive workload or poor-UX indications when an efficient personal style differs from the training cohort. Future studies should include broader anthropometric metadata, explicit inclusion criteria, and external validation before using such models in operational decision loops.
7. **Feature scope.** Only end-effector quaternion features were used in the predictive models. Inter-segment coordination (e.g., upper-arm–forearm coupling) and physiological signals (e.g., EMG, EDA) were not available but could provide complementary information channels.

## 6. Conclusions and Future Work

This paper provides evidence that the kinematics of an operator’s movement during IMU-based robotic teleoperation contain measurable and, for the majority of dimensions examined, statistically significant predictive signals about the operator’s subjective experience under a participant-level secondary-analysis design.

The key findings are as follows:

1. Movement entropy, higher-order moments, and kurtosis are reliable correlates of perceived workload and frustration, with Pearson and Spearman correlations reaching |r|=0.66 and |ρ|=0.68, respectively.
2. Target-specific regression models explain up to 78.7% of the variance in subjective usability ($R^{2}=0.787$), with seven out of 10 targets exceeding $R^{2}=0.50$. Nested permutation testing confirms statistical significance for 8 out of 10 regression targets.
3. Recasting the problem as binary classification yields AUC ≥0.75 for nine out of 10 targets, with three targets achieving perfect AUC. Permutation testing confirms significance for 8 out of 10 classification targets, supporting the feasibility of offline high/low workload discrimination from kinematics alone while not yet establishing deployment-ready real-time detection.
4. SHAP analysis [14] provides interpretable feature–target mappings that are consistent across both regression and classification tracks, with temporal irregularity and distributional shape features emerging as the dominant explanatory families.
5. These models are best interpreted as probabilistic indicators of UX and workload, not as measures of operator skill or objective competence.

The conclusions are consequently limited to the level directly supported by the results: statistically validated associations and held-out participant-level prediction for the significant targets. They do not imply causal mechanisms, universal generalization across operators, or operational readiness in closed-loop teleoperation. The two consistently weak targets, *physical_demand* and *intuitive*, should be treated as inconclusive in the present dataset rather than as successfully predicted dimensions.

Future work should prioritize the following:

- **Larger and more diverse samples** to increase statistical power, improve generalizability across gender, age, anthropometry, and experience levels, and enable explicit comparison with expert benchmarks;
- **Continuous in-task UX annotation** (e.g., per-trial ratings or physiological markers) to capture temporal dynamics and increase the effective sample size;
- **Multimodal data fusion** integrating physiological signals (EMG, EDA, heart rate) with kinematics for richer state estimation;
- **Real-time deployment** of the classifier in a closed-loop adaptive teleoperation system, closing the gap between offline analysis and online intervention.

## Figures and Tables

**Table 1:** Regression model families and hyperparameter grids used in model selection.

Model Family	Grid/Fixed Setup
LinearRegression	No tuned hyperparameters.
Ridge	alpha ∈{0.01,0.1,1.0,10.0}.
Lasso	alpha ∈{0.001,0.01,0.1}; fixed max_iter = 50,000.
ElasticNet	alpha ∈{0.01,0.1,1.0}, l1_ratio ∈{0.2,0.5,0.8}; fixed max_iter = 50,000.
KNeighborsRegressor	n_neighbors ∈{1,3,5}, weights ∈{uniform,distance}.
SVR	C ∈{0.1,1.0,10.0}, kernel ∈{linear,rbf}, gamma ∈{scale,auto}.
DecisionTreeRegressor	max_depth ∈{3,5,None}, min_samples_split ∈{2,4}, min_samples_leaf ∈{1,3,5}.
RandomForestRegressor	n_estimators ∈{100,200}, max_depth ∈{3,5,None}, min_samples_leaf ∈{1,3}.
GradientBoostingRegressor	n_estimators ∈{100,200}, learning_rate ∈{0.05,0.1}, max_depth ∈{2,3}, subsample ∈{0.8,1.0}.
XGBRegressor	n_estimators ∈{100,200}, learning_rate ∈{0.05,0.1}, max_depth ∈{3,5}, subsample ∈{0.8,1.0}, colsample_bytree ∈{0.8,1.0}; fixed objective = reg:squarederror, n_jobs = 1, verbosity = 0.

**Table 2:** Classification model families and hyperparameter grids used in model selection.

Model Family	Grid/Fixed Setup
LinearDiscriminantAnalysis	solver ∈{svd}.
RidgeLikeClassifier	C ∈{0.1,1.0,10.0}, penalty = l2, solver = liblinear; fixed max_iter = 20,000.
LassoLikeClassifier	C ∈{0.1,1.0,10.0}, penalty = l1, solver = liblinear; fixed max_iter = 20,000.
ElasticNetClassifier	C ∈{0.1,1.0,10.0}, penalty = elasticnet, solver = saga, l1_ratio ∈{0.2,0.5,0.8}; fixed max_iter = 20,000.
KNeighborsClassifier	n_neighbors ∈{1,3,5}, weights ∈{uniform,distance}.
SVC	C ∈{0.1,1.0,10.0}, kernel ∈{linear,rbf}, gamma ∈{scale,auto}; fixed probability = True.
DecisionTreeClassifier	max_depth ∈{3,5,None}, min_samples_split ∈{2,4}, min_samples_leaf ∈{1,3,5}.
RandomForestClassifier	n_estimators ∈{100,200}, max_depth ∈{3,5,None}, min_samples_leaf ∈{1,3}.
GradientBoostingClassifier	n_estimators ∈{100,200}, learning_rate ∈{0.05,0.1}, max_depth ∈{2,3}, subsample ∈{0.8,1.0}.
XGBClassifier	n_estimators ∈{100,200}, learning_rate ∈{0.05,0.1}, max_depth ∈{3,5}, subsample ∈{0.8,1.0}, colsample_bytree ∈{0.8,1.0}; fixed objective = binary:logistic, n_jobs = 1, verbosity = 0.

**Table 3:** Top joint Pearson–Spearman correlations for end-effector features.

Feature	Target	*r*	$p_{r}$	ρ	$p_{\rho }$
5th_moment_ee_quat.y	*effort*	+0.66	0.0056	+0.53	0.0348
entropy_ee_quat.x	*frustration*	+0.65	0.0070	+0.62	0.0101
mean_ee_quat.y	*temporal_demand*	−0.64	0.0080	−0.63	0.0084
4th_moment_ee_quat.x	*frustration*	+0.63	0.0093	+0.67	0.0045
variance_ee_quat.x	*frustration*	+0.62	0.0103	+0.68	0.0035
mean_ee_quat.w	*frustration*	−0.61	0.0123	−0.58	0.0183
5th_moment_ee_quat.x	*frustration*	+0.60	0.0132	+0.66	0.0059
std_peaks_ee_quat.x	*frustration*	−0.59	0.0153	−0.59	0.0161
kurtosis_ee_quat.x	*realistic*	+0.59	0.0165	+0.60	0.0142
entropy_ee_quat.x	*effort*	+0.59	0.0168	+0.59	0.0173

**Table 4:** Best target-specific regression configuration for each subjective dimension (LOSO).

Target	Model	Features	$R^{2}$	RMSE	MAE
*usability*	LinearRegression	x	0.787	0.335	0.262
*frustration*	LinearRegression	z+w	0.758	0.518	0.438
*effort*	Lasso	x + y	0.716	0.470	0.390
*realistic*	SVR	z	0.669	0.453	0.380
*performance*	LinearRegression	x + y + w	0.632	0.237	0.197
*mental_demand*	SVR	x + y	0.582	0.783	0.631
*temporal_demand*	Lasso	y+z	0.538	0.716	0.624
*responsiveness*	Lasso	x + y + w	0.374	0.740	0.574
*physical_demand*	DecisionTreeRegressor	*w*	0.005	0.897	0.792
*intuitive*	SVR	*w*	-0.087	0.966	0.855

**Table 5:** Permutation-test results for best target-specific regression models (nested protocol, $N_{\mathrm{perm}}=1000$).

Target	RMSE_obs_	RMSE_perm-mean_	*p*-Value	Significant
*mental_demand*	0.783	1.543	0.0010	Yes
*effort*	0.470	1.137	0.0010	Yes
*realistic*	0.453	0.919	0.0020	Yes
*performance*	0.237	0.749	0.0020	Yes
*frustration*	0.518	2.391	0.0040	Yes
*temporal_demand*	0.716	1.398	0.0050	Yes
*responsiveness*	0.740	1.304	0.0120	Yes
*usability*	0.335	6.496	0.0200	Yes
*physical_demand*	0.897	1.161	0.0779	No
*intuitive*	0.966	1.095	0.1558	No

**Table 6:** Best target-specific binary classifier for each subjective dimension (LOSO, median split).

Target	Model	Features	Acc.	Bal. Acc.	F1	AUC
*frustration*	LinearDiscriminantAnalysis	x+z	1.000	1.000	1.000	1.000
*performance*	LinearDiscriminantAnalysis	x+w	0.875	0.923	0.833	1.000
*usability*	LinearDiscriminantAnalysis	x	1.000	1.000	1.000	1.000
*mental_demand*	SVC	z+w	0.875	0.875	0.875	0.969
*responsiveness*	LassoLikeClassifier	x + y + w	0.813	0.833	0.812	0.968
*realistic*	LinearDiscriminantAnalysis	avg	0.875	0.867	0.867	0.933
*temporal_demand*	LinearDiscriminantAnalysis	w	0.875	0.855	0.855	0.927
*effort*	KNeighborsClassifier	x	0.875	0.857	0.867	0.921
*intuitive*	ElasticNetClassifier	z	0.750	0.500	0.429	0.771
*physical_demand*	LinearDiscriminantAnalysis	x	0.688	0.664	0.654	0.618

**Table 7:** Permutation-test results for best target-specific classification models (nested protocol, $N_{\mathrm{perm}}=1000$).

Target	AUC_obs_	AUC_perm-mean_	*p*-Value	Significant
*effort*	0.921	0.438	0.0030	Yes
*frustration*	1.000	0.428	0.0070	Yes
*responsiveness*	0.968	0.470	0.0080	Yes
*usability*	1.000	0.449	0.0130	Yes
*performance*	1.000	0.433	0.0140	Yes
*temporal_demand*	0.927	0.470	0.0180	Yes
*realistic*	0.933	0.480	0.0330	Yes
*mental_demand*	0.969	0.560	0.0400	Yes
*intuitive*	0.771	0.391	0.0699	No
*physical_demand*	0.618	0.449	0.1958	No

**Table 8:** Feature-set impact summary across tracks (means over best-per-target results).

Feature Set	${\bar{R^{2}}}_{\mathrm{best}}$	${\bar{\mathrm{AUC}}}_{\mathrm{best}}$
*Top* sets		
x + y	0.187	0.786
x + y + w	0.171	0.744
x + y + z + w	0.154	0.760
x + y + z	0.136	0.793
x	0.126	0.776
*Lower-performing sets*		
y+w	−0.107	0.678
w	−0.051	0.667
y+z+w	−0.044	0.688
z	−0.020	0.662
y	0.006	0.666

## Data Availability

The teleoperation dataset used in this study is described in [17] and made publicly available through the repository reported in [19]: https://github.com/eduardstan/authenticated-robotic-teleoperation, accessed on 2 August 2026. The analysis code has also been publicly released to support reproducibility.
